# If You Want the University to Change, Don't Theorise—Organise!

**DOI:** 10.1111/1468-4446.13200

**Published:** 2025-02-27

**Authors:** Sol Gamsu

**Affiliations:** ^1^ Durham University Durham UK

## Abstract

Organising as workers to build industrial power within our universities is a key element of how we respond to redundancies, marketisation and other political pressures on higher education. This piece argues that academics, as a subset of university workers, must interrogate their own working practices and commit to organising within their workplaces as a form of praxis. Practical steps of what organising might involve and long‐term strategic aims are identified drawing on the author's experience of organising within the University and Colleges Union in UK universities.

## 1 Introduction

If you want the university to change, then you are going to have to fight for it. If you want structural change, the fundamental rebalancing of power towards education workers and students, then prepare yourself, the path is long. And you are going to have things up along the way. I write this having spent three years (2021–2024) as branch president of my local branch of the University and Colleges Union (UCU), two of which were spent in national disputes and strikes. Most of these 3 years what I wanted was a stick of dynamite, what you actually have is a chisel.

## 2 (With)In, Against and Beyond the University

There is no way out of the decaying colonial, neoliberal morass of universities in our crisis‐riven societies which does not involve some transformation of how university workers, some of whom are ‘academics’, relate to our work. We cannot write, research, theorise or even teach our way out of this situation. We have to *organise* and commit to praxis, not simply the use of knowledge for the furtherance of our careers.

I write this because I believe knowledge is fundamentally out of shape. Most scholarship has very little to do with struggle, it is not ‘useful’ in that sense (Johnson 2007). Universities have been places where revolutionaries have learnt and revolutions have been fostered, but on balance they have mostly been institutions where the reproduction of power, capital and intersectional forms of violence have played out.

The university is a place where we can commit reparative theft (Joseph‐Salisbury and Connelly 2021), it is a place where anti or de‐colonial politics can be fostered, it is a place where class solidarities and gender norms can be both broken and re‐formed. We can do many of these things in this space by being in and against the institutions we work in (Mitchell et al. 1980), creating alternative spaces within institutions or elsewhere is important, but it is also not enough (Wilbert 2024), not if we are actually committed to trying to win rather than just make losing feel less bad.

Those of us who do this work often do so at the margins, as trojan horses within our universities. There are few things that have greater meaning, value, or joy than to help create the collective space and power for other people to organise, struggle and to learn about each other through that process. As someone committed to activist work, it has taken a long time to realise that perhaps the most important political act you can be involved in is to empower, politicise and engage others, helping them to form their own individual and collective consciousness of struggle and letting them take that forward. I believe that every picket, every general meeting, every conversation between workers is an opportunity for learning and education for political consciousness. This way of relating to knowledge and learning is quite different to what we are taught to do in the university.

Doing this work does come at a cost both personal and professional—you can't be the ideal neoliberal scholar churning out papers and grants and a militant union organiser/Palestine solidarity activist/housing campaigner. Our role within movements is not just to theorise them or sprinkle the intellectual gold dust of our analyses. This way of thinking about knowledge can only block the development of class consciousness and the formation of radical social movements (Castoriadis 1988). Intellectual and more importantly, *educative* work focussed on the formation of autonomous and collective forms of consciousness, not the individual display of intellectual or ‘academic’ prowess, is essential to political organising (Waugh 2022; Diniz‐Pereira 2013; Mirza and Reay 2000). Education and knowledge can be made to serve a fundamentally different, and revolutionary, purpose. Praxis requires theory and action. It requires us to theorise our action, to ask systematically, what do we need to do to defend ourselves and, ultimately, to win?

This work isn't easy. And it is harder to do if you are Black, a woman, trans, disabled, working‐class or precariously employed. If you are already an established, well‐published, well‐funded academic, you may want to consider whether prioritising organising within your university might be a greater contribution to higher education in the current moment.

We have to sneak this work under the radar and there are not enough of us, if we were ever in a position where this was at risk of becoming the dominant orientation of university workers towards their labour, what do you think Vice Chancellors would do? What would the state do? What would Capital do? These aren't entirely hypothetical questions, we have seen political repression in Indian or Turkish universities and, to a lesser degree, in UK universities during recent Palestine solidarity movements (Bhatty and Sundar 2020; Tutkal 2022).

For more people and more of the life on this planet to be able to survive and, one day, hopefully, thrive again, we need revolutionary, structural transformation. We need the end of capitalism and the end of imperialist, extractive and modernist logics of industrial growth. That may not mean a single cataclysmic revolutionary, change. It will probably mean non‐reformist reforms (Gorz 1967), organising here and now to create forms of dual power in communities (Food and Solidarity n.d.) and workplaces (Ness and Azzellini 2011) and defending each other against encroaching forms of fascism. But anything else is frankly not going to be sufficient at this point.

Are universities really set up for this? Are most academics willing to give up their commitment to bourgeois forms of knowledge? I hope that, given the chance, none of us would re‐create the university as an institution that uses knowledge to re‐create power, hierarchy, competition and destruction so systematically. Sometimes I do doubt how many would not re‐create it in deed if not in word, given the chance. More to the point, if we are serious about resisting then we have to think politically about our own workplaces, how we act, what we do in our everyday working lives but most of all how we can organise in our workplaces to build industrial power.

## 3 Unlearning (in) the University and Learning to Organise and Struggle

We *learnt* to be academics in this system, it took time and embodied practice. We learnt the ascetism, the passivity that is required to be a good student at school, to get good grades there, to achieve top grades in universities, to win PhD funding, to scrap it out for postdocs, teaching fellows and then finally, if we were lucky, to get a permanent post. Then we kept scrapping for funding and to make time to write our research so we could get promoted. When do we take a breath and ask what lessons we have been learning and whether these lessons are the right ones? You learn most things with time. Unlearning some of these lessons are essential to us winning. Unthinking or unlearning are ‘a means of both escape and self‐formation’ (Ball and Collet‐Sabe 2022: 994) but can only take place in a meaningful political sense through collective praxis (Means 2024).

We cannot defend ourselves without interrogating our own conditions of labour and how they compare to other university workers (Lê and Osserman 2021, 2022).‘Academics’ are the symbolically and, barring senior management, economically dominant workers within contemporary universities. The largest union in UK higher education, the UCU, is still dominated by academics. There are powerful and vocal Professional Services (PS) or Academic and Related Professionals (ARPS) members who often understand more of how the university actually works and is maintained. Some universities have large PS/ARPS membership than others or have more active branches of other unions (UNISON, UNITE and GMB) that organise administrative staff, porters, security guards, technicians, librarians in greater numbers. But in general these solidarities across workers are strong enough.

Why does this matter? Quite simply because if you want to shut down your workplace then you have to organise across all sections of workers in the institution. This is not just a question of changing how academics think about their own work and role, this is a strategic question of power and control over our workplaces.

## 4 Learning From the 2018–23 Strike Wave in UK Universities

In UK universities, the series of national strikes between 2018 and 2023 created an opening for more people to start to think differently about their working conditions.^1^ But not enough was done to overcome distinctions between workers or to create space for the political education required for this (Gamsu 2024). Too often strikes were by UCU alone, with the other higher education unions, and the workers they represent, absent. Stopping teaching or marking is one thing but if the library is still open, campus catering and accommodation is still functioning then the university is still running and management will seek to mitigate the effects of lost teaching.

The national strike of 2022‐23 over University Superannuation Scheme (USS) pension^2^ reform and the Four Fights (Pay, Casualisation, Workload and Equalities) highlighted at least three strategic issues that are important to current struggles. Firstly, questions of democracy, strategy and politics around the leadership of UCU caused major issues during the strike. The dispute highlighted consistent tensions between more militant branch activists and the union leadership of Jo Grady and the right of the union. Critcally, this included a decision by Grady and others to ignore the decision of UCU Congress, the annual conference of the union, to renew the strike mandate in a summer re‐ballot. This would have pushed the Marking and Assessment Boycott (MAB) into the new academic year which would have created severe pressure on management in institutions where the MAB was strong. In the end the reballot was delayed until after the end of the previous strike mandate so that staff were forced to do their unmarked work. The subsequent re‐ballot failed and the de‐moralisation of members and activists strengthened senior management's position. This national‐level demobilisation strengthened management's hand and laid the basis for the wave of redundancies that we have seen across universities in the UK since 2023. These questions of leadership and tensions between members and union bureaucracies/leaderships are an endemic issue within trades union history (Cohen 2014; Working Class History 2025).

Besides the question of leadership, there were two key weaknesses in the strike, unequal organising strength within institutions and between different institutions. I have already noted above, the issue with a strategy that primarily mobilises education workers in academic roles and neglects to organise other groups of workers within the university.

Secondly, the depth of organisation was uneven between universities which reflects the fact that organising in and against the university has to involve organising against the inequalities between universities. The pattern (which is not absolute) of the most privileged institutions having stronger union branches with greater resources and more stable financial positions is a problem. This is partly a result of action over USS pensions having built substantial experience of taking action, but it is important to understand that the financial position of different universities creates different terrain and stakes.

We must demand a redistribution of resources between universities. It is especially beholden on those of us who work in wealthier institutions that are more able to weather current financial pressures that we demand this change. The exalted, ideal conditions for the production of research that we are trained to desire require the maintenance of exploitative hierarchies within and between universities. It serves neither our strategic needs as workers seeking to organise across HE nor the needs for knowledge and education workers to be situated within wider projects of collective emancipation. And we all know the petty jealousies and real inequalities it creates within our own departments, closest to home. None of this is tenable.

Now, with universities implementing redundancies across large and mixed groups of workers (Queen Mary University of London UCU n.d.), the question of solidarity and collective class consciousness across job types is even more important. The questions that are raised here are pressing. You/we either get involved and get organising and organised or within very little time your university will look very different (if it doesn't already). I now want to turn to two questions, firstly what our ultimate aim and horizon for action is and secondly what tangible, practical things we should all be doing to build class consciousness and worker power within the university.

## 5 What Are We Aiming For?

Before talking about organising, let's set our goals. Our interest here is in dual power in the university. To paraphrase, then British Prime Minister, Lloyd George in 1919 (Cohen 2011: 59):

if a force arises in the university which is stronger than the university itself, then it must be ready to take on the functions of the university… are you ready?

I want to use this quote to illustrate two political goals. First, we have to aim for a level of political organisation where union strength and density such that there is no area of the university that could not be stopped by union members. Second, in building that strength we need to build the political consciousness that is capable of not only taking on but also undoing, re‐imagining and rebuilding all areas of the university whether it be cleaning or research support.

One of the issues that we face in organising in and against higher education is how people relate to universities. Compared to school workers, we do not have the same immediate political and public support and strategic pressure on the government to resolve strikes. It is far easier to damage the material conditions of students and university workers without it threatening the functioning of capital. A school strike removes defacto ‘childcare’, immediately creating problems for large numbers of workers. However, this is also about the enclosed and elitist nature of knowledge creation and the socially and academically selective nature of entry to universities. Both these tendencies make popular attachment to universities more difficult and underline the point that ‘a struggle in the university is always (or should always be) a struggle against the university as it is, and for the creation of something different’ (Gamsu 2024: 14). There is an extensive literature around alternative models of higher education and ways of learning (Maria, de Carvalho, and Mendes 2011; Moten and Harney 2004; Fuller 2018; Gamsu and Hall 2019). Incorporating the principles behind these models should be important to how we work and organise now. But rather than explore the detail of different models, I want to turn to the practical question of what organising in higher education might mean practically and how it aligns to a more long‐term set of political aims.

## 6 What Does Organising in the University Mean You Have to do?

Organising is not as complicated or as demanding as you might think. You might feel shy or unused to doing political work but you don't have to be. There are many forms of organising, from sending emails, building and maintaining databases, leafletting—political work isn't all giving speeches in front of crowds or meeting senior management. Building alternative forms of power, building relationships and collectives that are capable of resistance is the same as anything else, it has to be learnt and practised. Academics are just as (and no more) capable of this than any other worker. And just because you understand a system of oppression does not necessarily mean you know how to organise against it, sometimes it does and sometimes it doesn't. Begin from humility—there are no quick fixes, don't assume that your academic ability equates to political understanding within campaigns or organisations—if there were easy answers they would have been tried. We all have to learn about doing politics and to learn by doing.

Right now most universities in the UK are facing redundancies, several universities have live strike ballots and more will likely follow. So what should you practically do *now* to help strengthen the collective strength and class consciousness of your fellow university workers.

## 7 Practical Actions

1. Join a union.

In UK universities UCU is likely to be the largest and most active union.

2. Inform yourself about what is going on in your union. So:
  - *Read* your union emails, or make sure you check branch social media accounts
  - Attend branch meetings

3. Start talking about the redundancies and (any other) politics of work with your colleagues. This could mean:
  - Making time for a 5 minute conversation with a colleague about what is happening
  - If you work remotely, message or call a few colleagues.
  - Mention what is happening at the end of a work meeting.
  - Advertise forthcoming union meetings to colleagues. Inviting people directly is best.
  - Putting up posters, handing out leaflets around your area of work.
  - Avoid moaning without directing it towards some sort of action.

4. Organise a union meeting within your department or unit of work.

Creating a culture of union meetings within your local unit of work is *essential* to be building collective strength and worker consciousness. If you don't have a rep for your department, ask the branch to provide a list of members or to send an email on your behalf. If you can't do that, try and advertise a meeting—even if only a handful of you attend that is the seed for something bigger.

5. Volunteer. If you do some of the things under 3. then you are already volunteering. Being an active member is the first step. Ultimately, if you want to create a stronger basis for collective power for workers in universities, more of us will have to take on more roles. That might mean being on your branch committee (which can often involve as much or as little work as you want it to be bar regular meetings). But greater levels of activity or organising within your department could create roles there (imagine a regular union review of staff workloads within your department or departmental health and safety inspections of work‐related stress for example).

The practical steps align to a wider political set of aims that are important for wider organising with universities.

## 8 Long‐Term Political Aims

1. Creating a Collective, Political Consciousness Amongst Workers.
  This sometimes gets called, ‘engaging members’ and creating a participatory and democratic structure of unions is important. But it is not just a question of ‘engaging’ people but encouraging workers to gain their own political understanding of their working conditions and how they can organise to gain greater control and autonomy over their working lives.
2. Recruitment.
  In many parts of the university, workers will never have seen a union rep. This has to change. In person, targetted leafletting/campaigns are key. When doing this work, it is important to be conscious of gendered, classed and racialised divisions between workers. Increasing union density is not a magical bullet and it can sometimes be used to argue against action, as has been the case in UCU in recent years. Quite simply though, we need to organise in areas of the university that have not been organised. Strike waves also tend to see increases in members though this dynamic is complex (Cohen 2014).
3. Branch Structure and Organisation Matters.
  Ultimately, more people will need to take positions in branches. Often your local union branch will be kept going by a very small number of people who are over‐worked. This is not sustainable and limits what can be done. If you find yourself asking what ‘the union/branch’ is doing, you should also think about what you could *do*. Many of the formal roles are not as scary or demanding as you think, especially if more people are involved. Anyone who can send an email, leaflet or hold a brief conversation with a colleague can organise.
4. Formal Political Education.
  As I hope has been apparent here, there is a need for university workers to learn to think differently about their relationship to work. Some of this learning will come through struggle and fighting redundancies but there is also space for a more deliberate and structured approach to political education. We need a local, regional and wider national culture of education and time for learning and strategizing about how we win. Locally at Durham we are implementing regular away days for political education and planning. We will be having another one of these days the day before the annua ‘Big Meeting’ of trades unions, look out for an invitation. In London there have been a series of rank and file organising days. UCU's rep courses, the TUC/GFTU's courses and national initiatives like the Troublemakers Conference, the Ella Baker School for Organising are also worth attending (for links see below).^3^
5. Building Wider Union Structures.
  We need to build a wider political and community culture around trades unions. Trades councils bringing together different unions were essential to the 1926 General Strike. They retain this potential in places but have been considerably weakened. Re‐creating our trades council locally in Durham after the 2022‐23 strike wave meant that we organised a counter‐protest during the race riots of summer 2024. This sort of community‐based trades union work has to be extended and built out. Regional UCU structures and coordination between different universities and between universities and further education colleges, schools and community/prison education also needs developing.

These suggestions are my own notes for what needs to be done. There are many books on union organising for people to read (Allinson and Revolting 2022; McAlevey 2016; Little et al. 2023). We should, however, be wary of ‘gurus‘ rather than looking to histories of union militancy in the UK in the 1970s and current union movements across the Global South (Flanagan 2023). Accounts of smaller, class struggle/grassroots unions are important, especially as they have successfully organised outsourced, low‐paid workers in universities (Weghmann 2023). The University Worker Bulletins provide a workers' inquiry inspired approach to rank and file approach to understanding (The University Worker 2023; Woodcock 2014).^4^

As others have noted, strikes (Hodder and Mustchin 2024) and understanding class through a lens of exploitation and struggle (Toscano and Woodcock 2015) has been lacking in British sociology. Applying these questions to our own work and interrogating our own practical engagement in workplace struggle within the university has also been absent so this series in the *British Journal of Sociology* is important. I hope others will add their own ideas through this series and that these principles serve as a useful starting point for further discussion amongst sociologists and other university workers.

These discussions of our own political activity as workers in universities needs to be sustained. Our struggles whether over redundancies, insurgent fascism or climate change are urgent, but thinking about a long‐term political commitment is a necessity. Your contribution might be small but it must be consistent and sustained.

## 9 Hope in and for Universities Means Building Your Collective Power as Workers

If we want to defend ourselves, to defend universities, even as they are, and have the hope of making them into something different, then we need industrial power. We have to be able to shut down our universities, ideally all of them, simultaneously and completely. This is not going to happen overnight, but it does have to happen. We can keep wringing our hands and spilling elaborate sociological critiques onto the page but until we all start acting and organising nothing will change.

Education workers who work in higher education have to re‐orient our relationship to our own work and working conditions. Knowledge alone is not enough. Without praxis, the everyday nuts and bolts of organising work, we will get nowhere. No one is above that form of political work and everyone can organise in some form. The educational institutions we call universities need to be abolished and re‐created through wider struggles, but in the interim you have to think of yourselves as workers and as organisers and continue to ask yourselves what it takes to win and what we all have to *do*. There is no other source of collective hope for a better future in higher education and beyond. It will come from you and me as workers, working in solidarity with each other and students or it will not come.

No one else is coming to help.

## Data Availability

Data sharing is not applicable to this article as no new data were created or analysed in this study.

## Endnotes

^1^
There is an extensive Wikipedia entry on the 2018‐2023 strikes which provides a detailed overview of the disputes: https://en.wikipedia.org/wiki/2018%E2%80%932023_United_Kingdom_higher_education_strikes#September_2022_%E2%80%93_March_2023:_nationwide_strikes

^2^
USS is the pension scheme for universities established prior to 1992. The Teachers' Pension Scheme is the main pension scheme for universities that gained formal university status after 1992, the former polytechnic universities.

^3^

https://www.durhamminers.org/gala; https://www.ucu.org.uk/training; https://www.tuc.org.uk/training/TUCcourses; https://gftuet.org.uk/education; https://troublemakersat.work/; https://www.ellabakerorganising.org.uk/

^4^

https://notesfrombelow.org/author/university‐and‐college‐worker
